# Making intersectoral stakeholder engagement in medicine quality research work: lessons from the STARmeds study in Indonesia

**DOI:** 10.1186/s12961-025-01286-z

**Published:** 2025-02-19

**Authors:** Amalia Hasnida, Roland Bal, Reise Manninda, Stanley Saputra, Yunita Nugrahani, Faradiba Faradiba, Maarten Olivier Kok

**Affiliations:** 1 Department of Health Care Governance, Erasmus School of Health Policy and Management, Erasmus University Rotterdam, Rotterdam, The Netherlands; 2 Faculty of Pharmacy, Pancasila University, Jakarta, Indonesia; 3 Sydney School of Public Health, University of Sydney, Sydney, Australia; 4 Department of Health Sciences, VU University Amsterdam, Amsterdam, The Netherlands

**Keywords:** Medicine quality, Substandard drug, Falsified medicine, Knowledge translation, Collaborative governance, Stakeholder engagement, Intersectoral, Indonesia

## Abstract

**Background:**

Tackling falsified and substandard medicines requires intersectoral collaboration, impact-oriented research and the effective application of research findings. However, the best way to organize research and involve stakeholders from different sectors to ensure that results are used, remains unclear. We aimed to assess how intersectoral stakeholder engagement in research on medicine quality in Indonesia evolved, influenced the research processes and participants, and affected the uptake of the results.

**Methods:**

For this prospective case study, we adopted an abductive approach inspired by contribution mapping and collaborative governance. We conducted 37 interviews with key informants, observed 24 meetings and analysed 121 documents to systematically map the engagement of stakeholders in a study on medicine quality, focusing on processes, influences and research-related contributions.

**Results:**

From the outset, it proved feasible, but challenging, to effectively engage stakeholders in research into falsified and substandard medicines in Indonesia. After a cautious start and persistent efforts, stakeholders, such as the national medicine regulatory authority, became increasingly involved and developed a shared understanding of the need for intersectoral collaboration to tackle problems with medicine quality. While the research findings did not lead to a different estimate of the magnitude of the problem, the involvement of stakeholders was beneficial. After formalizing the collaboration, stakeholders provided data needed to study potential risk factors, product varieties and sales volumes, and contributed to decisions during the research and interpretation of the findings. Owing to frequent personnel changes and diverging priorities, stakeholder engagement required more effort than anticipated, and necessitated a strategic and adaptive approach. This approach had to account for the varying priorities and interests of stakeholders, the evolving framing of the problem, the implications of the findings and the nature of the field, where regulators must operate cautiously, balance interests and respond to critical incidents.

**Conclusions:**

Intersectoral stakeholder engagement in medicine quality research is challenging but beneficial. Engagement contributed to building trust and relationships between researchers and stakeholders, helped forge an intersectoral network focused on medicine quality, exposed the medicine regulator to new methods, inspired stakeholders to take on new roles and make better use of existing data and furthered a research–policy partnership forum on pharmaceutical topics.

**Supplementary Information:**

The online version contains supplementary material available at 10.1186/s12961-025-01286-z.

## Background

Substandard and falsified medicines represent a significant and growing threat to human health [1].

Poor-quality medicines can aggravate illness, leave patients uncured, and, in some cases, poison or kill people. Poor-quality medicines include substandard medicines, which are made by registered pharmaceutical companies, but do not meet quality standards; and falsified medicines, which are made, repackaged or sold by criminals who seek to deliberately misrepresent the identity, composition or source of the product [2].

Recent incidents involving medicine quality, such as the lethal cough syrup that killed hundreds of children in Indonesia, West Africa and India, underscore the critical importance of ensuring medicine quality [3]. While the attention to these tragedies might suggest that combating falsified and substandard medicines is a political priority, the reality is often different [4]. Despite lethal consequences, public outcry and media attention, sustained political commitment and resource allocation for addressing this issue remain insufficient, leaving significant gaps in the global pharmaceutical oversight framework [5].

Governments have long viewed the ensuring of medicine quality as a specialized technical task, primarily the responsibility of the national medicines regulator. The regulator sets standards for the production and distribution of medicines, and decides which products are allowed on the market. Manufacturers and distributors must follow these standards, implement quality control measures, and ensure the proper storage, transportation and handling of medications before they reach consumers. Regulators inspect and monitor compliance with these standards, as well as verify the quality of drugs circulating in the market. In practice, many national regulators are unable to fulfil their duties. In 2019, the World Health Organization (WHO) stated that “fewer than 30% of the world’s medicines regulatory authorities have the capacity to perform the functions required to ensure medicines, vaccines and other health products actually work and do not harm patients”[6].

Gaining insight into the prevalence of falsified and substandard medicines poses a significant challenge to regulators [2]. In most countries, thousands of authorized medicines, produced by both domestic and foreign manufacturers, move through complex supply chains. Meanwhile, many countries also contend with unauthorized medicines in circulation, as well as expired and falsified products. Although regulators are tasked with overseeing a complex market, they often lack the funding, facilities and specialized staff required to test medicine quality, and thus, gain little or no insight into the prevalence of falsified and substandard medicines [7, 8]. As long as the extent of the problem remains unknown, and its health impact remains invisible, issues with medicine quality will not feature prominently on the political agenda, and regulators will struggle to obtain sufficient funding.

Meanwhile, the limited data that are available offers a bleak picture. A recent review estimated that nearly 20% of antimalarials and over 12% of antibiotics in low- and middle-income countries are substandard or falsified [9]. Every year, the WHO receives hundreds of reports concerning suspected products, which are likely only the tip of the iceberg [2].

To more effectively combat poor quality medicines, experts have called for a more collaborative and research-based strategy [5, 10]. At the core of this new strategy is an understanding that problems with medicine quality are not just a technical issue that needs to be dealt with by the regulator, but are influenced by several risk factors that are deeply intertwined with the functioning of pharmaceutical markets, health systems and larger political and economic forces, and can only be addressed through a coordinated collective effort [5, 11]. An example of such a risk factor is a medicine stock-out, which pushes patients towards unregulated outlets, creating a market opportunity for those who sell fake products [12]. Another risk may emerge from procurement systems that push prices so low that companies are incentivized to produce substandard products [13]. To address these risks, the regulator needs to collaborate with others organizations, such as ministries of health, finance and trade, medicine producers and distributors, and law enforcement agencies.

A more effective approach to tackling poor-quality medicines also requires more impact-oriented research, and regulators who make better use of research findings [2, 5]. Regulators operate within a complex landscape of diverse goals and interests, which necessitates a careful, cautious and confidential approach [7]. This closed way of working hinders the sharing of valuable lessons and innovation. Academic researchers can help regulators by developing new methods and strategies for understanding medicine quality issues and creating and evaluating interventions.

A key aspect of impact-oriented research is stakeholder engagement. Numerous studies, in the health sector and beyond, have shown the benefits of engaging stakeholders in research: stakeholders can provide valuable knowledge and experience, help align research to local needs and circumstances, and improve its usefulness and legitimacy [14–17]. There is also evidence that engaging potential key users in research increases the likelihood that results will be used [15, 18].

While several evaluations point to the benefits of engaging stakeholders, recent studies have shown that it can actually be challenging to engage stakeholders in research [19]. Stakeholders may be uninterested or too busy, and constructively involving the right actors can require a lot of time and effort [20–22]. In Indonesia, it is standard practice to engage stakeholders during the dissemination of results, but intensive stakeholder involvement throughout the course of a study is uncommon [23].

In this article, we examine an attempt to combine an impact-oriented, engaged research approach with intersectoral collaboration in medicine quality research in Indonesia. There are three reasons why this medicine quality research was conducted in Indonesia. First, in 2016, the country experienced a widely-publicised case of vaccine falsification, which resulted in approximately 1500 children being injected with fake products [13]. This created a policy window to work on medicine quality. Second, the government had recently reformed pharmaceutical procurement and significantly pushed down prices, raising concerns regarding medicine quality [13, 24]. Third, Indonesia has a large domestic pharmaceutical market, with over 19 000 authorized medicines, and a relatively well-developed regulator who was interested in developing new methods to detect unsafe medicines [25]. The research team pursued an impact-oriented research strategy that required intersectoral stakeholder engagement. Throughout their project, the researchers sought collaboration with the regulator and other relevant stakeholders, anticipating that these partners would contribute to understanding issues with medicine quality, designing new methods and approaches, and interpreting and applying findings.

While there is a clear need for more impact-oriented research on falsified and substandard medicines, the best way to organize this research and engage stakeholders remains unclear. Insights into strategies for intersectoral stakeholder engagement could help to better organize research and apply the findings, thereby contributing to the fight against substandard and falsified medicines.

The aim of our study is to assess how intersectoral stakeholder engagement in research collaborations on medicine quality evolved and influenced research, people and organizations, as well as the uptake of the results. While we followed three interlinked research projects, this prospective analysis focuses on systematic tracking of at-risk medicines (STARmeds), the most recent project, which set out to estimate the prevalence of substandard and falsified medicines in Indonesia. We present data from interviews, observations and document analysis that show that intersectoral stakeholder engagement was feasible, yet challenging.

We argue that intersectoral engagement requires a significant effort and a strategic and adaptive approach, effective coordination and platforms for engagement, a careful framing of the problem, and attention to the nature of the field.

## Analytical framework

To guide our analysis of stakeholder engagement in medicine quality research, and illuminate its processes and influences, we draw upon insights from literature on knowledge translation and collaborative governance.

### Stakeholder engagement in impact-oriented research

Recent studies on research utilization provide an empirically grounded perspective on how research and engagement processes evolve and how results are translated into policy and practice [15, 17, 26]. These studies show that the use of research is influenced not only by the results and efforts of users but also by the embedding of research processes, the involvement of stakeholders in designing, conducting and interpreting the research, and developments in the broader context. These studies also show that the ideas that researchers formulate about how results will be used influence who they perceive to be stakeholders and how and when they engage these stakeholders [15, 27, 28].

These insights inspired our study of stakeholder engagement in research. Specifically, we:

1. Adopt a process perspective and analyse how research, embedded in a specific context, develops, progresses and gains meaning, as well as how efforts are made to apply the results.
2. Explore the ideas of researchers and other stakeholders about how results should be used, and how this itself shapes the stakeholder engagement strategy.
3. Examine how stakeholders are involved in the research process and how their involvement influences research activities and vice versa.
4. Analyse the influence of broader structures and dynamics in this context, including recent events and policy changes that affect the research activities and stakeholder engagement.

### Key elements in collaborative governance

An interesting aspect of the stakeholder engagement strategy in the STARmeds project is that it required intersectoral collaboration. Literature on collaborative governance provides inspiration for analysing how diverse organizations can work together to achieve a public goal [29]. According to literature, such intersectoral collaboration requires four key elements:

1. **A shared objective**: the willingness of diverse stakeholders to work towards a common goal.
2. **Effective coordination**: a neutral facilitator needs to coordinate the process.
3. **A forum for deliberation**: a platform for stakeholders to meet, discuss and make decisions.
4. **Inclusive participation**: engaging all stakeholders that are relevant to solving the problem.

Our prospective study focuses on stakeholder engagement in research. The four elements of collaborative governance provide inspiration specifically for our analysis of the intersectoral aspect of the stakeholder engagement.

## Methods

### Study design

For this prospective case study, we combined interviews, observations and document analysis to investigate how a research project and its intersectoral stakeholder engagement strategy evolved over time, and how different research-related contributions were realized. More details about our methodological approach are available in the supplementary material of consolidated criteria for reporting qualitative research (COREQ) reporting form (Supplementary Material 1).

### Analytical approach

Our analytical approach was inspired by contribution mapping, a method that is designed to analyse how research and translation processes evolve and are shaped by the actions of the researchers and those with whom they interact [15, 26, 27]. This approach focuses on how these processes evolve, and how they are shaped by historical developments, pre-existing networks and larger structures and dynamics in this context, and how these influence the uptake of the findings. As is common in contribution mapping, we focused on the actors involved in, or interacting with, the research project and the most likely key users in Indonesia.

A key part of contribution mapping is the development of a chronological process map, which contains a detailed analytical description of core activities, interactions and events that happen before, during and after a research project [27]. This three-phase project map provides a structure for data collection and analysis and for the presentation of the results. The chronological approach helps assess how actions, interactions and developments in the preparation and initial phase of a study influence how research and engagement processes evolve and how results are interpreted and taken up.

While our study focuses on the process of stakeholder engagement in the STARmeds project, we drew inspiration from literature on collaborative governance to analyse the intersectoral aspects of the stakeholder engagement strategy.

### The case

**Fig. 1:**
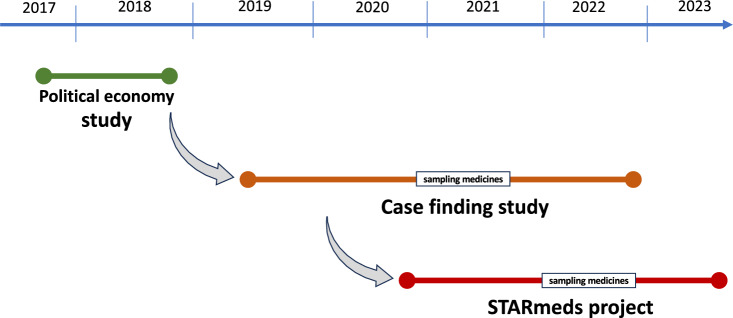
Timeline of different medicine quality studies in Indonesia

In this analysis, we focus on the stakeholder engagement in the STARmeds research project. This project was the third of three interlinked studies into medicine quality in Indonesia (Fig. 1). We chose the STARmeds project because it had the most comprehensive stakeholder engagement strategy, which included an intersectoral working group. One of the work packages of the STARmeds project focused on analysing the process of stakeholder engagements and how this shaped both the research and the uptake of the results. The current paper results from that part of the STARmeds study.

In the results section, we explain how the STARmeds project was developed upon the foundation of the two other projects (Fig. 1). As we will show in this paper, these previous engagements were formative in that they contributed to the underlying framings of the issues at hand and established relations with the stakeholders who were subsequently engaged in STARmeds, including the national medicine regulator, the Ministry of Health, the national health insurance agency and representatives from the pharmaceutical industry.

### Data collection

**Fig. 2:**
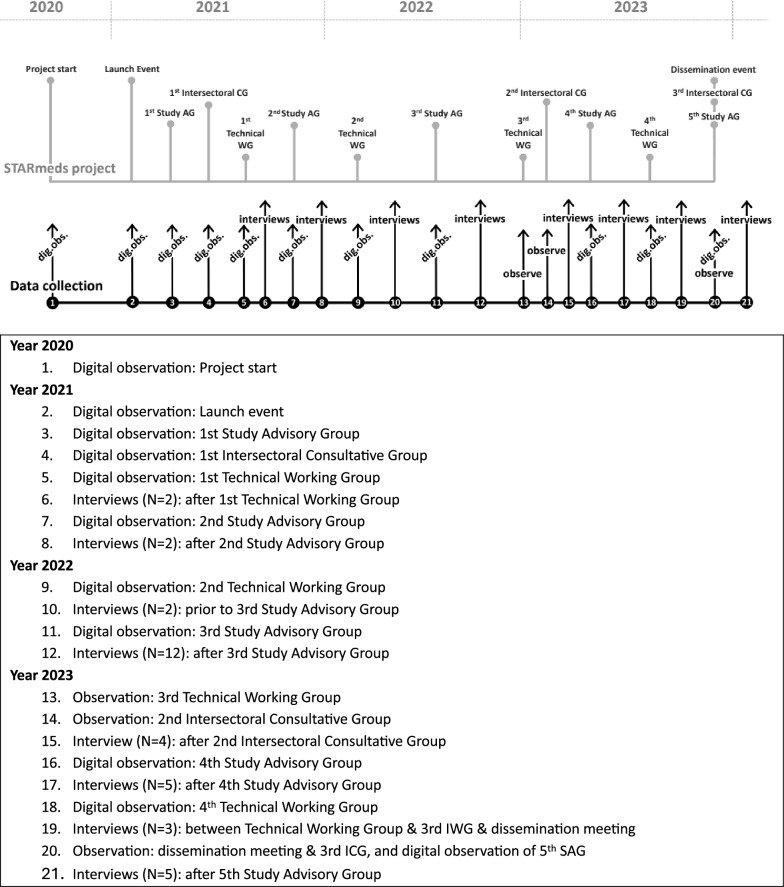
Timeline of data collection of policy learning study throughout the STARmeds project

Figure 2 provides an overview of the data collection during the STARmeds project.

#### Observing meetings

Between October 2020 and the end of 2023, we observed 24 meetings, with A.H. mostly acting as an observer. There were different types of meetings. For the intersectoral consultative group (ICG) (*n* = 3), the STARmeds team invited senior officials, key opinion leaders and decision-makers from various public, private and non-governmental organizations. The technical working group (TWG) meetings (*n* = 4) were organized to get technical input from stakeholders regarding data collection, analysis and interpretation. The study advisory group (SAG) meetings (*n* = 5) involved national and international experts and practitioners to advise on research plans and activities. There were also institutional audiences (*n* = 11) with particular organizations to discuss substantive topics, such as obtaining a research permit or interpreting laboratory testing results. At the end of the project, the researchers organized a large dissemination meeting. Meetings lasted on average 2 h. Owing to the coronavirus disease (COVID-19) pandemic, most meetings (18 out of 24) were held and observed online. Twenty-two (out of 24) meetings were audio-visually recorded; the recordings were not transcribed verbatim. Consent to record as part of the data collection was sought before the meetings started. Two meetings could not be recorded owing to the confidential nature of what was being discussed. We were, however, allowed to observe these meetings and made detailed notes. Our observations focused on the group dynamics between the STARmeds researchers and other stakeholders, as guided by contribution mapping and key elements of collaborative governance. For more information about key observational points, and other technical details, see the COREQ reporting form (Supplementary Material 1).

#### Interviews

To gain further insight into how the stakeholder engagement and research processes evolved, we conducted semi-structured interviews with 37 purposively selected key informants as detailed in Table 1. Interviewees were selected based upon their roles in the research, the stakeholder engagement activities and/or in the engaged organizations (e.g. SAG members, research teams, the medicine regulator, and the Ministry of Health).

**Table 1:** List of interviewees by roles

Roles	Frequency
Medicine regulator	9
Ministry of Health	3
National insurance	2
Pharmaceutical industry	4
Sub-national district authority	1
Procurement agency	1
Other ministries	4
World Health Organization	2
National research agency	1
Knowledge sector professional	1
Other study collaborators	4
STARmeds research team	5
**Total number of interviews**	37

We used an interview topic guide (Supplementary Material 2). Our topic guide was structured on the basis of contribution mapping and informed by theoretical concepts from collaborative governance, and adapted to the role and situation of the participant. We adopted a rolling triangulation approach, in which we used the data gathered from earlier interviews, observations and a documentary review to inform the creation of a specific schedule for each interview [30]. For more about the development of the topic guide, see the COREQ reporting form.

Interviews were structured chronologically based on the contribution mapping approach. First, we asked participants about their background and previous roles and responsibilities, and their perceptions regarding medicine quality. We continued by asking participants about their current roles and responsibilities, and perspective on medicine quality in Indonesia. We asked participants how they expected results should, and would, be used, and who and what would play a role in that process. We focused specifically on the engagement strategy and on how the engagement influenced the study and those who were engaged. Regarding the future, we discussed the potential uptake of the results, including any indications that results were informing policy and practice. Lastly, we asked participants to articulate what follow-up actions were needed in the light of the study findings, and who should initiate these actions. We recruited interviewees by approaching them by e-mail or in person during a project meeting. Two participants from the public sector did not respond to our interview requests.

As shown in Fig. 2, we conducted the interviews at various points during the research project.

Interviews were conducted both online and face-to-face, depending on the preference of the participants, research practicalities and the restrictions due to the COVID-19 pandemic. Interviews were conducted either in Indonesian (*n* = 29) or English (*n* = 8), and lasted 40 min on average. Thirty-six interviews were audio recorded. During the one interview that could not be recorded, the interviewer made detailed notes. A.H. led most of the interviews (*n* = 36); one interview was led by M.K.

#### Document analysis

To gain a deeper insight into the background of the project and triangulate our interview and observation data, we analysed a variety of documents produced throughout the STARmeds project (*n* = 121). We included documents which informed us about how the STARmeds study objectives were initially planned based on research proposals and amendments (*n* = 2), how the research design was changed and evolved following stakeholder engagement based on meeting minutes (*n* = 95), and ultimately realized and publicized based on periodic activity reports (*n* = 17), as well as project publications in the media and scientific journals (*n* = 6). We also included one field note from the political economy and risk-flagging study to understand how stakeholder engagement during these two projects shaped the initial STARmeds research design. We excluded internal meeting minutes which were specifically about operations and managerial aspects.

### Data analysis

Interviews were transcribed verbatim. Data analysis started with developing a general timeline of the case study, including the research activities, stakeholder engagement processes and influence of events in this context. This general timeline was structured according to the three-phase process map (before the start of STARmeds, during STARmeds and after the results were finalized). We used an abductive approach and thematic analysis to identify patterns of information emerging from the data [31]. We developed a coding tree by performing open coding followed by axial coding [32]. During three workshops, A.H., M.K., R.M., S.S., Y.N. and F.F. read multiple sets of interview transcripts and highlighted important information. Emerging themes were discussed during the plenary sessions and we agreed on a first list of codes. Next, during the axial coding steps, the codes were refined by coding another set of transcripts. Our coding tree is included in Supplementary Material 3. We then applied these codes to all transcripts, observation notes and documents for analysis.

We then linked the emerging themes to the process map to present our results in a detailed chronological narrative. Between these steps, we organized several sessions between A.H., R.B. and M.K. to discuss emergent theoretical themes, leading to the use of collaborative governance theory to enrich the analysis. We used ATLAS 24.2 as qualitative data analysis software. For more information about data analysis, see the COREQ reporting form.

## Results

We present the results chronologically in three consecutive phases: (1) the research and policy context prior to the study and during proposal development, (2) the conduct of the STARmeds study, and (3) the finalization and dissemination of results. In each phase, we examine how research activities and engagement processes evolved, the role of stakeholders and the influence of contextual structures and dynamics.

### Setting the stage: medicine quality incidents in Indonesia

In 2016, Indonesia was rocked by the discovery of fake measles vaccines given to children for over a decade, propelling the issue of medicine quality to the top of the political agenda, and prompting a major overhaul of the national medicine regulator. The following year, a multinational research team, coordinated from Erasmus University Rotterdam, began investigating the political and economic factors driving the proliferation of falsified and substandard medicines in middle-income countries [11–13, 33]. The developments in Indonesia were highly relevant. Besides the scandal with fake vaccines, there were also worries about substandard medicines entering the regulated supply chain. The Indonesian government had reformed procurement policies, resulting in significantly reduced medicine prices [24]. In various forums, manufacturers, distributors and patients voiced concerns that these low prices might compromise product quality.

An Indonesian researcher on the multinational team investigated the root causes of a fake vaccine scandal. Her analysis revealed that unmet patient demand created an opportunity for criminals to sell falsified vaccines [13]. In addition, she found that procurement systems, by pushing prices excessively low, could incentivize companies to cut corners, potentially resulting in substandard medicines. After presenting their findings in 2018, the team initiated a follow-up study to identify which medicines were at the highest risk of poor quality. They aimed to test whether market-risk indicators – such as low prices or company history – could reliably predict medicine quality. Developing these indicators required detailed data on the pharmaceutical market and regulatory inspections. To conduct the study, the researchers collaborated with scholars from a university in Jakarta who had experience studying medicine prices in Indonesia and a strong working relationship with the national drug regulator [24]. Using the risk indicators, the researchers planned to send mystery shoppers to three regions in Indonesia to buy specific products, which would then be quality-tested in a lab.

### How the engagement evolved in previous research

During the initial two studies, the research team actively engaged stakeholders, expecting them to provide valuable data and insights that would enhance the research’s relevance and increase the chance of its results being applied. The researchers focused on engaging the national medicine regulator. The researchers presented their proposals and conducted a formal meeting with the regulator, discussing the uncertainties surrounding the prevalence of poor-quality medicines in Indonesia. The regulator expressed interest in new methods for post-market surveillance and agreed to future meetings. Engagement with the regulator continued in 2019 as part of the risk-flagging study.

### Developing the STARmeds research proposal

While the risk-flagging study was ongoing, the research team submitted a proposal to a United Kingdom (UK) funder for a larger study on the prevalence of poor-quality medicines in Indonesia. This new study, called STARmeds, was led by a principal investigator from Imperial College London, in collaboration with researchers from Universitas Pancasila in Indonesia and Erasmus University in the Netherlands. The primary aim of STARmeds was to estimate the prevalence of substandard and falsified medicines in Indonesia, as well as to assess the societal costs associated with poor-quality medicines. STARmeds was designed based on the groundwork of the risk-flagging project, building upon the same team, network and sampling locations.

#### Planning intersectoral stakeholder engagement

From the beginning, stakeholder engagement was ingrained in the research strategy. The researchers had designed a specific engagement strategy with three components. First, the project would establish an intersectoral consultative group, with representatives from public, private and non-governmental organizations who would contribute data, knowledge and experience to jointly develop the new prevalence estimation method. Second, the researchers envisioned an institutionalized partnership between academia and policymakers, which would be convened by an Indonesian ministry with an overarching coordinating role. Third, to foster collaboration, the researchers proposed to conduct part of the work from the regulator’s office.

During the first two studies, the researchers had learned that engaging the regulator was not a straightforward process. One of the challenges was that the medicine regulator had a distinct role within the system, necessitating independence, confidentiality and strict adherence to formal procedures. One of the researchers explained:

*Regulators should follow the strict line of authority…[…]… they have a different mindset, while researchers are way more flexible. *(notes from observations)

### Preparing to implement STARmeds during COVID-19

In May 2020, the researchers learned that the STARmeds project was approved. Meanwhile, the world had drastically changed. The COVID-19 pandemic had reached Indonesia, significantly impacting research planning and stakeholder engagement. The regulator was occupied with evaluating and authorizing pandemic-related medical products, leaving little time for joint activities. In-person collaboration at the regulator’s office was no longer feasible, and all stakeholder engagement had to shift online.

While the regulator was busy with COVID-19, the researchers wanted to secure institutional support from the regulator. To guide this effort, they enlisted a former chief medicine regulator as an advisor. Securing institutional commitment for collaboration proved challenging owing to rapid turnover of senior staff, organizational fragmentation and concerns about data usage.

*We had to present (the research design) in many directorates. It was not only to the post-market surveillance directorate but also to the research directorate, to high-level decision makers…[…]… But sometimes the people changed, then we started presenting again…[…]… In the beginning we put the (proposal) letter with the name (of the official) but when we followed up two weeks later, the people were changed. After that we just put the name of the directorates (on the letter).* (researcher)

### Forging a network and building trust

As Indonesia entered lockdown, the research team started to implement its stakeholder engagement strategy. A first challenge was to develop a shared commitment among diverse stakeholders and gather their input for refining the study design. The researchers aimed to convene stakeholders in an intersectoral working group hosted by a government organization. However, the initial response from stakeholders revealed that fostering collaboration towards a common goal was not easily accomplished.

*One of the challenges […] the silos between institutions and even within institutions. You know between deputy X and deputy whatever. It’s kind of a complete silo effect. You know that sectoral ego thing is huge, particularly in this space. You’ve got civil war basically between the regulator and ministry… Within the ministry between the different divisions. Then you’ve got the procurement agency struggling with the ministry about sectoral versus national. Then, on top of that, the insurance agency doesn’t speak to the ministry. And then, oh, the regulator won’t speak to the industry at all.* (researcher)

Given the differing perspectives and priorities among stakeholders, and with many preoccupied by the COVID-19 pandemic, the researchers decided to adapt their engagement strategy. The researchers decided to serve as conveners for the intersectoral consultative meetings, using their status as neutral outsiders to facilitate collaboration.

During the online intersectoral meeting, senior staff from key ministries and other government organizations attended, and a member of parliament delivered remarks in support of the project.

*The theme of this research is currently an ongoing discussion (in parliament). The role of medicines in the Indonesian health system is very vital, especially as we have seen so far during this pandemic. It is important to provide a rational dosage of medicines to patients and it is also equally important to ensure the safety and quality of medicines on the market.* (notes from observation)

Formalizing the collaboration with the regulator proved more challenging than anticipated. While the regulator maintained interest in the project, it became increasingly clear that they saw the study not only as an opportunity but also as an added task and potential risk. Some officials expressed concerns that the study could serve as an evaluation of their performance, while others questioned how to structure the collaboration to avoid the risk of the regulators becoming mere data suppliers. The researchers reassured the regulators that the study was not intended to evaluate their work and promised to discuss findings before making them public. Data confidentiality emerged as another concern, resulting in lengthy negotiations over contracts and multiple meetings and presentations.

*Again and again, like more than five times. Until we were very bored. Oh my god, it’s again and again. And even though there are no changes, a new person came in and asked again similar things and then sometimes they were very vocal. They say it’s not possible, it’s a big change. But we already agreed in the previous meetings. But the new person came and said: ‘This is a no. Not possible’. And then, fortunately, some person from the old discussions explained to them that we already discussed this in the previous meeting.* (researcher)

The regulator also expressed concern about the workload involved in managing the data that the researchers had requested to develop the risk indicators. To avoid overburdening the regulator, the researchers agreed that they would handle the bulk of the data management and analysis. A senior official explained that the trust that had been built during previous interactions with the Indonesian scholars proved instrumental in addressing these concerns and fostering the collaboration.

*I have trust…[…]…Trust in the study leader also influenced me. So trust grows from the people we already know.* (regulator)

While the formal agreements laid the groundwork for data sharing, the responsibility for the data within the regulator’s large organization was less defined. Multiple departments were involved in managing key datasets, raising questions about roles and responsibilities and necessitating internal coordination. Some data requests that the researchers anticipated to be simple, proved to be complex for the regulator, resulting in lengthy waiting periods.

### Engaging stakeholders in selecting which medicines to sample

The researchers engaged the Ministry of Health and other key stakeholders in designing the sampling strategy. A crucial decision was determining which of the thousands of medicines on the market would be included in the study. With a budget sufficient to collect and test approximately 1200 samples, the researchers planned to select five or six different medicines. Different stakeholders proposed different criteria for selecting the medicines. The researchers focused on public health importance, risk groups and the feasibility of collecting samples. The Ministry of Health was concerned about antimicrobial resistance and proposed the inclusion of antibiotics. The regulator advised medicines with a record of abuse, such as tramadol. After careful deliberation, the researchers decided to include five prescription-only medicines: amoxicillin, amlodipine, cefixime, allopurinol and dexamethasone.

### Changing the research design

In October 2021, after numerous meetings and negotiations, the formal agreements between the universities and the regulator were finally concluded, and the researchers gained access to existing inspection data that had been collected by the regulator. Using the data from the regulator, the researchers started to model and test their ideas about the risk categories, and soon found that there was no clear association between the risk-indicators and the results of previous inspections. The researchers presented these findings during a technical working group with the regulator and decided to modify their study design. Instead of trying out a risk-based sentinel surveillance approach, the researchers opted for a sampling strategy that focused on price variation, as this was thought to be the most likely risk factor influencing the quality of medicines.

### Another incident influencing stakeholder engagement

In early 2022, while the researchers had started collecting data, another major incident with dangerous medicines emerged, propelling the issue of medicine quality to the top of the political agenda once again and influencing stakeholder engagement [34]. Several Indonesian manufacturers failed to test a medicine’s raw ingredients and used a highly toxic chemical to produce children’s cough syrup. The contaminated syrup caused acute kidney failure, killing over 200 children. This tragic case demanded the full attention of the regulator, limiting its ability to participate in other research. The incident, however, did underscore the importance of medicine quality and inspired other stakeholders to participate more actively in the STARmeds study.

### Deliberation on the estimation methods

While data collection for the STARmeds study was ongoing, two other medicine quality studies in Indonesia reported their findings [35]. These studies had tested medicines that were also included in the STARmeds study. Both studies found that the prevalence of substandard medicine was low and there was no relation between the price and quality of the medicines.

Once the laboratory testing results became available, the STARmeds team began estimating the national prevalence of falsified and substandard medicines in Indonesia. During the risk-flagging study, the researchers had obtained data on medicine sales volumes in the Indonesian market, which showed that many common medicines had numerous product varieties with diverse market shares. If a product with a large market share was found to be substandard, it would impact many more patients than a product with a smaller sales volume. Therefore, the researchers proposed incorporating sales volume data into their prevalence estimates.

Meanwhile, stakeholders in the technical working group began questioning the goal of estimating the nationwide prevalence of poor-quality medicines. The primary concern, raised by the regulator and other stakeholders, was that the researchers planned to present a national prevalence estimate based on a study that included only five types of medicines, despite thousands being authorized for the Indonesian market.

*My real concern is that the systems thinking is good, but the data is very limited. So, if the data is only for five medicines, then we assume (quality) per product types, for example amoxicillin tablets … […]… But if the quality of all medicines is estimated (nationwide), where does the data come from? We need more data to conclude that. The most sensible thing is to only make estimates of the prevalence of these five types of medicines.* (manufacturer)

### A changing perspective on the role of stakeholders

Once the initial results became available, the dynamics of the stakeholder engagement process shifted. The researchers observed that several stakeholders became more active in the discussions.

*I remember when we had our very first working group where it was like you were talking into a big black hole, there were no questions, there was no engagement, there was nothing…[…]… and I think last week when we had the technical working group there was more engagement. I could see that. I think it had also to do with the fact that…[…]… maybe beforehand they couldn’t quite imagine what kind of research would look like that we are producing. And I think that it wasn’t until it was on paper that they understood what this is about. They kind of saw the value of it.* (researcher)

The researchers’ perspectives on the stakeholders had also evolved. After nearly 2 years of interactions, the researchers had gained a deeper understanding of the stakeholders’ roles, responsibilities and ways of working. Initially, the researchers focused primarily on the regulator, expecting it to be the key user of the study results. While some staff expressed interest, the regulator remained unconvinced about the necessity of a new prevalence estimation method and continued to adopt a formal and risk-averse approach to collaboration.

Meanwhile, the researchers began to recognize the strategic role that the Ministry of National Planning (MNP) could play in utilizing the findings. Responsible for overseeing key development indicators, the MNP was interested in the study’s results, as the prevalence estimate provided an independent validation of the regulator’s performance.

The researchers also reconsidered their approach to engaging industry. Despite recommendations from other stakeholders, the research team had opted against involving industry from the start, fearing it would hamper the engagement of the regulator. This lack of early engagement led to issues in the project’s second year when the researchers sought industry assistance to confirm medication packaging for identifying fake products. Industry representatives were taken aback by the request, as they had received minimal information about the study, and many did not respond.

### Finalizing the results and disseminating them to stakeholders

Despite scepticism from some stakeholders, the researchers proceeded with their plan to calculate a national prevalence estimate. On the basis of 1274 tested samples, they estimated that the prevalence of substandard medicines in Indonesia was 4.4%. Although the researchers employed a different method, their estimate did not differ much from the regulator’s routine inspection results, which reported a prevalence of 4% in 2022.

In October 2023, the results were presented at a large dissemination meeting attended by a diverse group of stakeholders. During the presentation, the researchers compared their prevalence estimate with the regulator’s inspection results, concluding that post-market surveillance was effectively implemented in Indonesia. At the meeting, senior staff from the regulator responded positively to the study results, expressing relief that the findings aligned closely with their own inspection results. During a follow-up interview, the regulator adopted a more sceptical stance, continuing to question the validity of a nationwide prevalence estimate based on a sample that included only five different types of medicines.

Another finding highlighted by the researchers was that there was no relationship between the price and quality of medicine. This finding meant that the government’s efforts to reduce the prices of publicly procured medicines did not compromise their quality. This conclusion drew critical comments from industry representatives, who argued that the researchers had only assessed the active pharmaceutical ingredients. They pointed to the recent cough syrup case, where many children died due to the poor quality of excipients – an aspect not tested in the STARmeds project [3].

A second finding was the ease of purchasing prescription-only medicines from unlicensed outlets. The researchers pointed out that many Indonesians obtain their medicines from unlicensed outlets. In routine inspections, the regulator only checked to see whether the products that were illicitly sold were falsified, but did not assess their quality. The researchers recommended to test for substandard products as well. During the follow-up interviews, the regulator remained critical of this idea, and argued that it was not part of its mandate to ensure the quality of medicine that was sold illegally.

The STARmeds project had also resulted in outputs that could be used in other countries, including a method for estimating the cost of sampling and testing, and a toolkit [36]. A regulatory expert highlighted the possibilities of these methods, but warned that applying them elsewhere would not be easy.

*Certainly, countries should be able to apply this methodology. But.…[…]… having academics carrying this (methods) out is one thing. Having a regulator is another, who has a range of other duties. So, I think there’s a good argument there for engaging with academic institutions.* (regulatory expert)

### Intersectoral collaboration as output

While there were no indications that the findings were directly applied in Indonesia, the study did result in the formation of an intersectoral network focused on medicine quality. Stakeholders noted that their involvement in the study made them aware that ensuring medicine quality is not solely the responsibility of the regulator, but is also influenced by other organizations.

*As a person who procures goods and services, I now feel I am responsible for this. Why? Because the medicines purchased by the government follow our rules. Is it sufficient to use market authorization as a preliminary quality assurance? So far, we have never made specific criteria for the quality of medicine that must be listed on the procurement platform. So far, we assumed that if a market authorization has been issued, it means the drug is fit for distribution. (procurement official)*

Several stakeholders praised the collaboration between the study and government organizations. A regulator staff member emphasized that this experience increased their awareness of the benefits of partnering with academia and other sectors to address medicine quality issues. One of them explained:

*We did not realize what potential (work) can be maximized by joining forces with other stakeholders.*

During the last intersectoral meeting, researchers asked stakeholders about the future of the intersectoral forum following the study. Stakeholders expressed interest in continuing the platform, suggesting it be broadened to include pharmaceutical policy and access to medicines [37].

While participants agreed to continue the forum, there was uncertainty about who should organize it. Researchers advocated for government leadership, while some stakeholders proposed that the regulator take charge. Others suggested that the Ministry of National Planning serve as the overarching coordinator. A staff member from the MNP proposed to convene a meeting to discuss this plan and to organize a first meeting in 2024.

## Discussion

The aim of our study was to assess how intersectoral stakeholder engagement in medicine quality research evolved, influenced research processes and participants, and affected the uptake of results. Our findings show that while engaging stakeholders was challenging, it proved beneficial. Stakeholders provided valuable data and insights that informed the research process. They were introduced to new ideas, methods and roles, becoming integral members of an intersectoral network focused on medicine quality. However, engaging stakeholders was labour-intensive and required an adaptive and strategic approach, taking into account the diverse priorities and interests of stakeholders, as well as the cautious nature of the field.

Our analysis provides insights into the efforts and strategies required for effective stakeholder engagement in impact-oriented research. Our results show that stakeholder engagement requires considerable effort and substantial time and dedication [19, 22]. Rapid turnover of staff and organizational changes among stakeholders can further complicate engagement, necessitating persistence, patience and a willingness to continually build relationships with new staff. Our analysis highlights the importance of formalizing collaborations and aligning them with institutional goals to reduce dependence on individual support [38].

Previous research highlights the role of trust in effective engagement of stakeholders [14, 15]. We found that stakeholder engagement benefitted significantly from personal relationships and trust developed during previous projects, as well as the strategic involvement of trusted individuals, such as a former director of a key stakeholder organization. Moreover, as actors frequently change, trust-building is a continuous effort. These findings underscore the importance of investing in long-term partnerships that extend beyond a single project, and in building relationships with both individuals and institutions [39, 40]. Developing procedural arrangements, such as memorandums of understanding, can help sustain collaborations during transitions in personnel.

We found that a lot of efforts necessary to engage stakeholders and establish effective collaboration did not take place during formal meetings, but rather behind the scenes. While much of the work for organizing the formal meetings could be handled by junior staff and an engagement manager, senior researchers, who held significant respect and trust, had to engage in informal discussions before and after meetings, which was key to making the collaborations work. The need for both front-stage and backstage work has also been highlighted in other studies [41]. We would add that effective coordination is essential between activities conducted in front of and behind the scenes, as well as among the various individuals involved.

Our analysis shows that stakeholder engagement requires a strategic approach. The first element of this strategic approach is the development of a framing of the research that is suitable for gaining support and commitment from stakeholders. To develop an appealing framing, the research team explained to each stakeholder how they could contribute to tackling an important problem, while also taking into account the different needs, concerns and interests of the individual stakeholders. This actor-specific framing occurred, for example, when the researchers sought to alleviate the regulator’s concerns by emphasizing that the project should not be viewed as an evaluation of the regulator, but as an opportunity to jointly develop new methods and approaches.

The second element of this strategic approach is to carefully consider who should be engaged, when and for what purpose. Whereas literature about collaborative governance suggests that stakeholder identification is something that should be done at the start of a project, we found that this is actually a continuous undertaking, and dependent on evolving ideas about the meaning of the results, how results could be used and who should play a role in that process [14].

Our analysis also shows that one should be aware of the limits of stakeholder engagement. In the STARmeds project, the research team initially focused on the regulator as the potential key user and engaged them from the start. At their preference, the researchers involved the industry only towards the end of the study. While this may have helped secure the regulator’s engagement, it ultimately complicated the process of confirming sample packaging. This finding aligns with other studies that emphasize the importance of carefully considering who is involved and when, as there are trade-offs and a limited number of stakeholders that can be actively engaged [19]. In future research, experimenting with strategies to involve a more diverse array of stakeholders, such as civil society and community representatives, may be beneficial.

We also found that researchers need to be flexible in order to benefit from the input from the stakeholders. The research team that we observed had planned a study in which they could incorporate the input of the stakeholders, for instance in the selection of medicines. The input of the stakeholders, including the data that they provided, inspired the researchers to make significant changes to their study design. While in this case study the research funder agreed with this change in research design, this flexibility is not always provided. Other studies report similar adaptations that are made as a result of the input of stakeholders, and suggest that these adaptations help align research to needs, and increase the likelihood that results will be used [15].

We observed that, in relation to this flexibility, there was also a willingness of the research team to adapt to the needs of stakeholders, by giving updates and presenting some preliminary results or providing some analytical support. While these efforts may have sometimes distracted the researchers from their core tasks, they helped forge the collaboration. This finding suggests that it can be helpful to allocate some dedicated resources for stakeholder engagement and arrange for operational flexibility in the planning of research [14].

According to literature, inter-organizational collaboration to achieve a public goal needs a forum for deliberation and coordination by a relatively neutral facilitator [29]. In the STARmeds project, there were multiple platforms for engagement. The most ambitious platform was the intersectoral consultative group, in which a variety of stakeholders was brought together, who had to be convinced that they could play a role in better understanding and tackling problems with falsified and substandard medicine. The researchers organized and facilitated these meetings, which had both advantages and disadvantages. The researchers took on the role of convenor of the intersectoral meetings after they became aware of the difficult relationships and lack of cooperation between some of the organizations that played a role in the field of medicine quality. The researchers were eager to make the collaboration work, had a dedicated budget for the engagement, and had a relatively neutral position which allowed them to navigate the politically charged topic and coordinate the process. We found that the meetings facilitated the collaboration of diverse stakeholders, fostered a shared framework for addressing medicine quality issues, and helped develop and strengthen relationships.

Having academics organize such meetings also has disadvantages. As relative outsiders to policy processes, they have little formal authority, which can make it more difficult to involve high-level decision makers. A second disadvantage is their dependence on project funding, which can cause carefully built partnerships to disintegrate when a project concludes. In Indonesia, there seems to be sufficient interest among those involved to continue the platform, and by broadening the subject there seems to be a good chance that this collaboration will continue.

We found no indications that the research results were directly applied in Indonesia. This is not unusual, as studies of research use suggest that results are often not applied immediately, but only over time, in a more conceptual way, and contribute to change through a more cumulative process [42, 43]. We did find that the stakeholder engagement led to several relevant developments and beneficial outcomes. One key outcome was the formation of an intersectoral network of organizations that recognized their role in combating poor-quality medicines in Indonesia. At the heart of this network was a shared understanding that market dynamics, such as product shortages, price pressures and financing flows, could influence the production, distribution and consumption of poor-quality medicine. This suggests that the engaged research strategy succeeded in reframing the problem of medicine quality and fostering collaboration, which are crucial steps towards protecting patients from ineffective and unsafe products and ensuring that the medicines they receive work as intended.

Based upon our analysis, we have formulated 12 suggestions for intersectoral stakeholder engagement, which are presented in Table 2.

**Table 2:** Practical recommendations for intersectoral stakeholder engagement

• **Recognize that effective stakeholder engagement is a continuous process requiring substantial effort and resources**. Ensure the allocation of adequate resources – such as time, funding and personnel – to support engagement activities
• **Map key stakeholders and strategically plan when and how to engage them**. Recognize that stakeholders have distinct roles and vary in influence and interest. Anticipate the potential consequences of each engagement choice, as engaging with one stakeholder can impact your ability to engage others
• **Engage stakeholders from the start**. Involve stakeholders early in the research process to collaboratively shape priorities, objectives and methodologies. This early engagement fosters a sense of ownership and promotes long-term commitment to the project
• **Introduce the study and engagement process thoughtfully**. Tailor the approach and framing to align with each stakeholder’s needs and priorities. Providing clear terms of reference helps set expectations and build trust
• **Jointly develop a shared vision that aligns the goals of different stakeholders**. A shared vision helps bridge differences in priorities and enables stakeholders to work together towards mutually beneficial outcomes
•** Acknowledge that stakeholders need time to become familiar with the research topic, the engagement process and their potential contributions**. Providing training can enhance their ability to contribute meaningfully, allowing them to grow into their roles over time
• **Build relationships with individual stakeholders and their organizations**. Staff turnover may necessitate engaging new individuals; therefore, formalizing agreements with organizations can help sustain progress and ensure continuity during staff changes
•** Ensure flexibility in project planning and stakeholder engagement**. Emerging challenges and unexpected results may alter stakeholders’ roles. Be prepared to adapt the research and engagement strategies based on shifting priorities, new insights and changing problem framings
• **Build trust by transparent communication, active listening and delivering on promises**. Clarify roles, responsibilities and expectations. Share information regularly, respect diverse perspectives, interests and concerns, and consistently follow through on commitments
• **Keep stakeholders engaged throughout the project**. Provide regular progress updates on milestones and developments, showcase quick wins and encourage stakeholders to ask questions and provide feedback
• **Monitor the engagement processes and adapt as necessary**. Solicit feedback from stakeholders and reflect on the process to enhance engagement efforts
• **Develop a long-term stakeholder engagement strategy**. Build meaningful relationships with stakeholders beyond the scope of a single project and plan continuous engagement activities

### Strengths and weaknesses

Prospectively studying stakeholder engagement in research has both strengths and weaknesses. By conducting a prospective study, we were able to closely monitor and comprehensively capture the changing dynamics of involvement in a multi-year project. However, as resonating with earlier work, this study is quite interdependent [19] with the progress of the research project that we studied. We would have liked to interview some stakeholders more often. This was not always possible, because stakeholders were also expected to provide input to the STARmeds project and we did not want to overload them with multiple research-related requests in the same period. Our strategy then was to triangulate the information obtained from observations with interviews and document analysis. Although the participants were aware of the fact that our research team observed them as part of stakeholder engagement in research, some still asked us about our opinions regarding this process and the interpretations of the STARmeds study findings, which we politely declined to answer. They nonetheless indicated that they were not disturbed by our presence and their awareness of it did not influence our findings.

## Conclusions

Tackling substandard and falsified medicines requires collaboration, impact-oriented research and the effective application of results. Our study demonstrates that intersectoral stakeholder engagement in medicine quality research is challenging yet beneficial. Engagement helped build trust and relationships between researchers and stakeholders, forged a diverse network of organizations committed to combating poor-quality medicines, exposed the medicine regulator to new methods, inspired stakeholders to adopt new roles and better utilize existing data, and advanced a research-policy partnership forum on pharmaceutical topics. However, making intersectoral stakeholder engagement effective posed challenges: it required sustained efforts from a dedicated team, a strategic approach, careful framing of which actors to involve, and thoughtful consideration of when and how to engage. Meaningful engagement also necessitated operational flexibility to seize opportunities and adapt plans based on stakeholder input and contextual changes.

## Supplementary Information

Supplementary Material 1

Supplementary Material 2

Supplementary Material 3

## Data Availability

The datasets generated and/or analysed during the current study are not publicly available owing to privacy provisions. At times, the interviews that underlie this study discuss confidential issues. During our informed consent procedure, we assured participants of anonymity. We are unable to comply with that commitment if we share the full interview transcripts. Researchers are welcome to request specific coding queries by contacting the corresponding author.

## Abbreviations

ICG: Intersectoral consultative group
MNP: Ministry of National Planning
SAG: Study advisory group
STARmeds: Systematic tracking of at-risk medicines
TWG: Technical working group
