# Supplementary material for: Making intersectoral stakeholder engagement in medicine quality research work: lessons from the STARmeds study in Indonesia
Source: Health Res Policy Syst. 2025 Feb 19;23:21. doi: 10.1186/s12961-025-01286-z (PMC11840975; doi:10.1186/s12961-025-01286-z)
Supplement: Supplementary file 1 — Supplementary Material 1 [file 12961_2025_1286_MOESM1_ESM.docx]

Supplementary 1. Consolidated Criteria for Reporting Qualitative Research (COREQ)

| **Domain 1** |  |
| --- | --- |
|  |  |
| *Personal Characteristics* |  |
| 1. Interviewer/Facilitator | Amalia Hasnida (with in some interviews participation from MK, RM, YN, SS, and FF – for details see the answer to question 7) |
| 1. Credentials | MSc |
| 1. Occupation | Researcher |
| 1. Gender | Female |
| 1. Experience and Training | International Public Health and Healthcare Governance  The researchers have experience in quantitative and qualitative research in a variety of areas including medicine quality, healthcare governance and knowledge translation. |
|  |  |
| *Relationship with participants* |  |
| 1. Relationship established | The researchers who conducted the interviews, AH, with sometimes others joining in, had no prior relationships with the majority of the study interviewees and participants in the observations.  Several interviewees (n=4) had collaborated with AH on previous research projects including on medicine quality. |
| 1. Participant knowledge of the interviewer and observer | The interviewers, AH with others sometimes joining in, introduced themselves and their backgrounds, and provided detailed information about their interest in the research topic when they approached study participants about the possibility of an interview, and again prior to the interview. Interviews were mostly led by AH (n=36); one interview was lead by MK. RM, YN, SS, and FF joined in for 11 interviews and introduced their roles before the interview started.  Before observing participants at various meetings (e.g., intersectoral consultative group, technical working group, Study Advisory Group meetings, individual audiences), the participants were given an introduction to the policy learning study as part of the larger *Systematic Tracking of At-Risk Medicines* (STARmeds) study by the co-principal investigators including the observation that would take place during the meetings. AH also reintroduced the objective of policy learning study when asking several follow-up questions to participants individually during breaks or after the meeting. Although the participants were aware of the fact that our research team observed them as part of stakeholder engagement in research, some still asked us about our opinions on this process and the interpretations of the STARmeds study findings, which we politely declined to answer, but which indicated that they were not disturbed by our presence.  AH led almost all observations (n=22) except two face-to-face meetings in Jakarta. FF, RM, YN, and SS also contributed to take detailed field notes under the guidance of AH. |
| 1. Interviewer characteristics | The interviewers had no financial or non-academic interest in the field. |
|  |  |
| **Domain 2** |  |
|  |  |
| *Theoretical Framework* |  |
| 1. Methodological orientation and Theory | This prospective study follows the stakeholder engagements in a research project to develop a method for estimating the prevalence of substandard and falsified medicines in Indonesia (The STARmeds project).  We draw upon insights from the contribution mapping method and collaborative governance to follow the stakeholder engagement process and to analyse the formation of collaborative networks between the researchers and other stakeholders in the STARmeds project and how study results were received by stakeholders.  We used an abductive approach in which we drew upon contribution mapping process map to structure our interview topic list and observation points and informed by theoretical concepts of collaborative governance specifically for our analysis of the intersectoral aspect of the stakeholder engagement. Throughout the study, we iteratively adjusted the focus of the interviews, observations, and document analysis based on insights we gained from the initial data collection phase.  s |
|  |  |
| *Participant Selection* |  |
| 1. Sampling (for interviews, participant observations, and document analysis) | We attended all meetings organized by the STARmeds study team (i.e., intersectoral consultative group, technical working group, Study Advisory Group, and institutional audiences). We excluded meetings with regional or district authorities for the sole purpose of obtaining research permit for fieldwork as they were not engaged by the research team during the STARmeds project to develop methods for estimating the prevalence of substandard and falsified (SF) medicines. In addition, we excluded meetings between the STARmeds research team and the study collaborators e.g., market data provider and third-party laboratory as these meetings addressed operational aspects of the project and did not specifically about stakeholder engagements.  The study interviewees were first identified based on their roles and contributions in various meetings within the STARmeds study (e.g., intersectoral consultative group, technical working group, Study Advisory Group meetings). We adopted “a rolling triangulation” approach in which we used data collected from earlier interviews, observations, and documentary review, to inform the creation of specific schedule for interviews (Methods, Interviews).  We analyzed various documents that informed us about the development and evolution of the STARmeds study objectives, research design, stakeholder engagement strategies, and final study results. (Methods, Document analysis). |
| 1. Method of approach (for interviews) | Interviewees were approached either in person during one of the STARmeds meetings or via institutional request letters for interviews, emails, and phone calls. The interviewees were informed about the study aim, the reasons for our particular interest in interviewing them, and the measures taken to protect confidentiality. |
| 1. Sample size (for interviews, participant observations, and document analysis) | A total of thirty-seven (n=37) study participants were formally interviewed for the study. (Methods, Interviews)  We observed 24 meetings held during the STARmeds project including the intersectoral consultative group or ICG (n=3), Technical Working Group (TWG) (n=4), Study Advisory Group (SAG) (n=5), institutional audiences (n=11), and a study dissemination meeting (n=1). (Methods, Observing meetings)  We analyzed a total of 121 documents. We included initial research proposals and amendment for the study funders and stakeholders (n=2), periodic activity reports for stakeholders (n=17), minutes of weekly coordination team meetings and meetings with external stakeholders (n=95), and project publications in the media or scientific journals (n=6). We also analyzed one field note from two previous medicine quality projects prior to STARmeds (i.e., the political economy and the risk-flagging study). We excluded internal research team meeting minutes which were specifically about operations and managerial aspects of the project (Methods, Document analysis)  We also presented and sought feedback on research design and initial findings from the Study Advisory Group (SAG) members and their comments informed our data analysis. (Methods, Data analysis) |
| 1. Non-participation (for interviews) | Of the thirty-nine (n=39) individuals contacted, two (n=2) from the public sector did not respond any further after we sent an institutional request, and eventually did not participate in this study. (Methods, Interviews) |
|  |  |
| *Setting* |  |
| 1. Setting of data collection (for interviews and participant observations) | The interviewees that agreed were then interviewed face-to-face (n=7), and other participants who were unable to meet in person were interviewed by phone (n=1) or video conferencing platform (n=29).  All interviews were conducted at the participants’ working hours (n=37). We interviewed a small number of participants in their workplaces (n=2), and at places chosen by the participants e.g., participants’ homes or restaurants near their workplaces (n=5).  Most meetings (n=18) were observed online due to the circumstances during the COVID-19 pandemic. Offline meetings (n=6) were conducted in the convention meeting rooms and the participants’ workplaces in Jakarta. (Methods, Observing meetings) |
| 1. Presence of non-participants (for interviews) | Only the study participants and the researchers were present during the interviews. |
| 1. Description of sample (for interviews and observations) | We aimed to include a wide variety of interviewees based on their roles in the STARmeds research, stakeholder engagement activities and/or in the engaged organizations (e.g., SAG members, research teams, the medicine regulator, Ministry of Health). Table 1 in the paper provides more detail. (Methods, Interviews)  For observations, we included meetings with stakeholders organized by the STARmeds research team (n=13) as well as institutional audiences (n=11). We observed institutional audiences with the medicine regulators (n=6), the Ministry of Health (n=2), the Statistics Indonesia (n=2), and another ministry (n=1). |
|  |  |
| *Data Collection* |  |
| 1. Interview guide (and observation guides) | We conducted semi-structured interviews. The interview guide was structured following contribution mapping and informed with theoretical perspectives on collaborative governance specifically for our analysis of the intersectoral aspect of the stakeholder engagement, and tailored to the role or profession of each interviewee. At the beginning of the interview guide, we asked about their previous roles and responsibilities, as well as their perspectives on medicine quality in Indonesia. We particularly used insights from contribution mapping to identify the researchers’ scenarios about the research process, engagement strategies, and to understand the potential for uptake including indications that the results were informing policy and practice. (Methods, Interviews)  Focusing on intersectoral engagement strategy and collaborative network formation, we structured our discussion to inquire about the interviewees’ perceptions on how intersectoral collaborative forums were organized in the STARmeds studies, the capacity for joint actions between research team and stakeholders, and factors that facilitated and hindered the collaboration.  The interview guide was not pilot tested. We discussed the interview guide and its use during our team meetings, and some modifications were made after the initial interviews, especially when the results of the STARmeds study were known.  Our observations focused on the group dynamics and interactions between the STARmeds researchers and other stakeholders during the various meetings in the STARmeds study. On the one hand, we were guided by contribution mapping approach when observing the relevant actors and key users among the STARmeds research team and stakeholders, for example which stakeholders actively contributed to the discussions, who shaped the discussions including making decisions or forming consensus. Moreover, we paid considerable attention to the interactions between the STARmeds research team and stakeholders to jointly shape the research design, interpret the study results and discuss practical and policy implications.  On the other hand, we used key elements of collaborative governance to observe how an intersectoral forum of deliberation was established (e.g., how it fosters dialogue between stakeholders), how effective coordination processes were maintained in the forum (e.g., in managing the process, resolving disagreements, guiding discussions), how the exchange of views between the research team and stakeholders occurred in formulating and agreeing on a shared objective (e.g., what kind of framing were used, what policy topics were linked and discussed related to the STARmeds’ study goals), and how inclusive participation was achieved among stakeholders and the research team (e.g., which stakeholders were invited, which stakeholders did not attend meetings, etc).  Our observation points were iteratively adapted throughout the course of the STARmeds study based on the discussions with our research team. |
| 1. Repeat interviews | No repeated interviews were carried out. |
| 1. Audio/visual recording (for interviews and observations) | The majority of the interviews (n=36) were audio-recorded with the consent of the study participants. One interviewee declined to be audio recorded. In that case, the interviewer took detailed written notes during the interview. (Methods, Interviews)  We obtained written informed consent, and repeated consent on tape for those consenting to be audio-recorded. (Methods, Data collection). Interviews were anonymized and both interviews and consent files stored off-line.  Almost all meetings were visually recorded except for the meetings (n=2) because of confidentiality concerns. When no recordings were made, we were allowed to observe and took detailed notes. (Methods, Observing meetings)  Consent to record as part of the data collection was sought before the meeting started, particularly in the case of online meetings via video conferencing platforms. |
| 1. Field notes (for interviews and observations) | In addition to notes taken during the interviews, field notes about contextual factors (e.g., setting, non-verbal expressions of the interviewee, etc.) prior, during or after the interviews were kept in a logbook to assist the interpretation of the interviews.  We also made detailed observational notes of the meetings in our field notes as guided by several key observational points based on contribution mapping and collaborative governance. Additionally, we recorded several dimensions of observation such as the physical space or place, layout of the meetings, actors, activities, objects that were being discussed (e.g., regulation or technical guidelines), time, purpose of the meetings.  AH consolidated all observational notes by FF, YN, RM, and SS into the same document for analysis. The findings and interpretations were discussed as a group when necessary. |
| 1. Duration (for interviews and observations) | Interviews were conducted in the participant's choice of Indonesian (n=29) or English (n=8), and lasted an average of 40 minutes. (Methods, Interviews)  Participant observations were conducted in English (n=8) and Indonesian (n=16) based on the language used in the meeting. The meetings lasted an average of two hours. (Methods, Observing meetings) |
| 1. Data saturation | We tried to include a wide variety of interviewees from various roles and professions to be able to corroborate the information between them and thus to achieve data saturation. The collected data was then compared with the information derived from participant observations and document analysis. Then, the insights were discussed within the study team as data collection progressed.  For some categories of interviewees, we interviewed only one key participant. However, based on data triangulation with participant observations and document analysis, we are confident about the validity and reliability of our study findings. (Discussion, Strengths and weaknesses) |
| 1. Transcripts returned | Interview transcripts were not returned to participants.  Participants observations were not transcribed verbatim. We analysed the data using observation field notes and reviewed the recordings when necessary. |
|  |  |

| **Domain 3** |  |
| --- | --- |
| *Data analysis* |  |
| 1. Number of data coders | Our team developed a coding tree by performing open coding followed by axial coding. During three workshops, AH, MK, RM, SS, YN and FF read multiple sets of interview transcripts that felt particularly rich for initial coding and highlighted important information. The coding-approaches among the researchers were discussed, until consensus was reached. (Methods, Data analysis) |
| 1. Description of the coding tree (for interviews, observations, and document analysis) | A coding tree is included as an additional file. We used this coding tree for interviews, observations, and document analysis. |
| 1. Derivation of themes (for interviews, participant observations, and document analysis) | Emerging themes were discussed during plenary coding sessions and our team agreed on a first list of codes. Subsequently, during axial coding steps, the codes were refined by coding another set of transcripts. Between these steps, emerging themes and analysis were discussed between AH, RB, and MK, leading to the use of collaborative governance theory. The initial coding tree was then revised iteratively as the work progressed. (Methods, Data analysis). |
| 1. Software (for interviews, participant observations, and document analysis) | ATLAS.ti 24.2. was used as qualitative data analysis software. |
| 1. Participant checking | While we did not share transcripts with the interviewees to avoid influencing their pre-recorded thoughts and opinions, we did conduct member checks or participant validation among the investigators to appropriately interpret the data. We also shared our key preliminary findings with several study participants, which facilitated additional perspectives which informed our final analysis and interpretation. |
| 1. Reflexivity | Our team, which consisted of researchers from Indonesia and the Netherlands, engaged in reflexive discussions during data collection and analysis to examine how our positionalities shaped our research. The data collection was primarily conducted by Indonesian researchers who had a relevant background (pharmaceutical science and public health) and who had previously been involved in health care systems and/or pharamaceutical research in Indonesia. The prominent role of the Indonesian scholars was crucial for truly understanding the case and gaining the trust of other researchers involved in the STARmeds project and other stakeholders in Indonesia. The researchers from the Netherlands contributed by providing advice on theory and methodology and joining critical reflection, which helped to ensure the rigor of our research. |
| *Reporting* |  |
| 1. Quotations presented (for interviews and observations) | Quotations are presented to illustrate the findings. The quotes are provided in the main paper and are categorised by participants’ roles or professions. We primarily provide quotes from interviews because we are mainly interested in participants’ interpretations of stakeholder engagement in research and intersectoral collaborative networks. |
| 1. Data and findings consistent | There was consistency between the data presented and the findings. |
| 1. Clarity of major themes | This study aimed to assess how intersectoral stakeholder engagement in research on medicine quality evolved, influenced research processes and participants and the uptake of the results. The first most prominent theme was about the challenges of stakeholder engagement and the strategies to overcome them in the STARmeds study. Next  were actors’ expectations about the research process and study results as well as their interpretations of the disseminated findings. Another prominent theme was about the roles and responsibilities of actors, for example, which actors can be potential key users of the study results and who should participate in the intersectoral consultative forum on research about substandard and falsified medicines. |
| 1. Clarity of minor themes | While study participants reflected on stakeholder engagement challenges in research during the STARmeds study, we identified less prominent themes of engagement facilitators and best practices from their previous working experiences in research or practice. Furthermore, although we have probed for this theme, we did not gain much insight into how the main STARmeds study results (i.e., the method for estimating the prevalence of substandard and falsified medicines) would be used by key potential users. |
