# Supplementary material for: Making intersectoral stakeholder engagement in medicine quality research work: lessons from the STARmeds study in Indonesia
Source: Health Res Policy Syst. 2025 Feb 19;23:21. doi: 10.1186/s12961-025-01286-z (PMC11840975; doi:10.1186/s12961-025-01286-z)
Supplement: Supplementary file 2 — Supplementary Material 2 [file 12961_2025_1286_MOESM2_ESM.docx]

Supplementary 2. Interview topic list

**Notes:**

This is an indicative list of topics intended to guide the interviews in general. Questions relevant to external stakeholders other than the STARmeds research team are annotated.

## **Part I: The Past (roles and responsibilities, initial expectations)**

1. What are your overall roles and responsibilities in your institution?

- What are your overall roles and responsibilities in your institution regarding substandard and falsified (SF) medicines?

1. In recent years, have SF medicines played some role in your work or been an issue on the agenda?

- If yes, what was the related agendas?
- If yes, what are the roles?

***Initial expectations about the STARmeds study***

1. What are your views on the quality of medicines in Indonesia in general? *(for stakeholders other than the STARmeds research team)*

- In your opinion, what are the current issues related to substandard and falsified (SF) medicines in Indonesia?
- Do you have a particular medicine/patient population/locations/area that you are concerned about?

1. What were your expectations about the findings at the outset of the project?

- How did you envision the main findings of STARmeds?

1. At the outset of the project, which actors and institutions do you think would benefit most from the study?

- Why? What roles do they have that you think make them influential actors?
- How do you envision the results of the study being used by specific actors in Indonesia and/or other countries? Who did you expect to be involved?
- How do you think the results of the study will be used in other countries?

## **Part II: The Present (evolving expectations, stakeholder engagement, ICG, institutional roles)**

***Evolving expectations about the STARmeds study***

1. What do you think are the main findings of the STARmeds study?

- What are the most unexpected findings?
- What evidence gaps do you think these findings could address?

1. How did your initial expectations about the study results evolved evolve?

- What factors play a role in shaping the study?
- What aspects do you think met your expectations? Why?
- What aspects do you think did not meet your expectations? Why?

***Stakeholder engagement***

1. How was your experience of engaging various stakeholders in the STARmeds study?

- Did you find it added value to the research process?

--- If yes, what were the most valuable aspects of the engagements?

--- If no, how would you change or improve the current process?

- What specific things did you learn from these interactions?

1. Which stakeholders do you think are:

- Easier to approach or engage?
- Quite challenging to approach or engage?
- What are some strategies for engaging these challenging stakeholders?

1. What do you think are the facilitators and barriers to engaging stakeholders?
2. Are there any stakeholders that you think should have been engaged or more involved more during the STARmeds study but have not been so far?

- What roles and responsibilities do these stakeholders have regarding SF medicines or medicine quality?

***Intersectoral consultative group (ICG/PEMO)***

1. How was your experience the process of Intersectoral Consultative Group (ICG)/PEMO or the technical working group?

- Did you learn anything new from the meeting?

--- If yes, what was it?

--- If no, why?

- Did it change your opinion on SF medicines issues in Indonesia?

---If yes, how?

1. In your opinion, what are the facilitators and barriers in the ICG or PEMO platforms or technical working group?

- What are the strategies to overcome these barriers?

1. In your opinion, what are the areas for improvement in the ICG/PEMO meetings?

- What are the reasons?
- How can these points for improvements be realized?

1. Are there other institutions that should play a role in addressing SF medicines that have not been invited to PEMO?

- If yes, what are these institutions?
- If yes, what role can these institutions play?

1. Have you had any previous experience with a research partnership model?

- If yes, for what purpose? When and with whom?
- If yes, how is the current ICG or technical working group meeting different from previous meetings?

1. After the Intersectoral Consultative Group (ICG) or the technical working group meeting, have you done anything with the insights or information at your institution? *(for stakeholders other than the STARmeds research team)*

- If yes, how was the process?

--- To what extent did ICG or technical working group meetings help put SF medicines on the agenda?

1. After participating in the Intersectoral Consultative Group (ICG) or technical working group, do you think the research team is sufficiently informed about the problems of SF medicines or its prevalence estimates? *(for stakeholders other than the STARmeds research team)*

- If no, what aspects or insights might have been missed?

## **Part III: The Future (results uptake, scenario of roles and implications, stakeholder engagement)**

***Results uptake***

1. What are your expectations regarding the uptake of the final STARmeds study results?
2. To what extent are the results currently being used?
3. Which institutions do you think will be the main users of the results or findings of this study?

- What are the reasons?
- Are there any unexpected stakeholders that you think need to know about the results of STARmeds study?

1. How do you think the results of this study will be applied to relevant users or stakeholders?

- What resources needs to be available to users to implement or use the prevalence estimatation method developed by STARmeds?

***Policy implications and scenario of roles***

1. What policy and regulatory changes might you expect based on the STARmeds findings?

- At what level are these policies in place or being implemented (sub-national/national/regional/global)?
- Do you expect any policy changes outside the pharmaceutical or health sectors?

1. Which agencies should act on the results of the STARmeds study?

- Why?

1. Do you think yourself as an academic as having a role in acting on the results of the STARmeds study?

- If yes, what kind of role?
- If no, why not?

1. Do you see yourself or your institution as having a role in it? *(for stakeholders other than the STARmeds research team)*

- If yes, what kind of role?
- If no, why not?

***Stakeholder engagement & intersectoral consultative group (PEMO)***

1. To what extent do you think stakeholder engagements and Intersectoral Consultative Group (ICG) can be useful or beneficial for academic research projects?
