# Supplementary material for: Making intersectoral stakeholder engagement in medicine quality research work: lessons from the STARmeds study in Indonesia
Source: Health Res Policy Syst. 2025 Feb 19;23:21. doi: 10.1186/s12961-025-01286-z (PMC11840975; doi:10.1186/s12961-025-01286-z)
Supplement: Supplementary file 3 — Supplementary Material 3 [file 12961_2025_1286_MOESM3_ESM.docx]

Supplementary 3. Coding tree

| **First-order concepts (examples)** | **Second-order concepts** | **Aggregate themes** |
| --- | --- | --- |
| Rapid turnover of the senior staff in the engaged organization | Engagement challenges | Evolution of intersectoral stakeholder engagement in research |
| Fragmented structure of the engaged organization |  |  |
| Concerns about data confidentiality |  |  |
| Contextual factors e.g., COVID-19 pandemic hinders in person engagement and coordination |  |  |
| Built trust due to collaboration in previous projects | Engagement drivers |  |
| Flexibility and adaptive approach of the research team in engaging the stakeholders |  |  |
| Contextual factors e.g., COVID-19 pandemic drives the stakeholders to take part in tackling substandard and falsified medicines |  |  |
| Careful framing of research aims to achieve shared commitments among actors | Engagement strategies including forming an intersectoral collaborative network |  |
| Considerations on which actors to involve, timing, purpose, and arrange effective coordination (e.g., engaging the industry towards the end of the project) |  |  |
| Establishment of an intersectoral consultative group convened by the researchers |  |  |
| Selecting the study medicine based on public health importance | Informing the research design and analytical interpretation of results | Influence of stakeholder engagement to research process |
| Changing the research focus from sentinel surveillance to sampling based on price variation |  |  |
| Changing the researchers’ ideas about the importance of post-market and good manufacturing practice based on study implications |  |  |
| Building trust and relationships between researchers and stakeholders to work towards a common goal | Forging an intersectoral network |  |
| Ministry of National Planning (MNP) as one of the key potential users | Changing the perspectives about other strategic stakeholders in using the findings |  |
| Creating the awareness of substandard and falsified medicines issues | Mutual learning | Influence of stakeholder engagement to research participants |
| Exposing the medicine regulator to new methods of estimating the prevalence of substandard and falsified medicines |  |  |
| Inspiring stakeholders to take new roles in tackling substandard and falsified medicines | More active participation of stakeholders in intersectoral meetings |  |
| Making better use of existing data |  |  |
| Medicine regulator was relieved that the prevalence estimation result is similar with their routine inspection | Substantive research output | Influence of stakeholder engagement to the uptake of study results |
| Inspiring the use of the study results in other country settings |  |  |
| Initiative to further intersectoral research-policy partnerships forum beyond the STARmeds study | Intersectoral collaboration or relational output |  |
| Suggesting the public sector as the next potential meeting convenor |  |  |
